# Persistent Zinc Deficiency Among Residents of a Long-Term Care Facility: A Five-Year Longitudinal Study

**DOI:** 10.7759/cureus.107317

**Published:** 2026-04-18

**Authors:** Norifumi Kudeken

**Affiliations:** 1 Internal Medicine, Kubagawa Medical Clinic, Naha, JPN

**Keywords:** frailty, longitudinal study, long-term care facility, older adults, zinc deficiency

## Abstract

Background

Zinc deficiency is common in older adults, particularly among residents of long-term care facilities, yet its longitudinal course in this setting remains insufficiently described.

Methods

We conducted a five-year longitudinal observational study using routine health examination data collected between 2021 and 2025 in a long-term care facility in Okinawa, Japan. Serum zinc concentrations were measured annually at the same clinical laboratory. Residents with at least two zinc measurements were included. Zinc deficiency was defined as a serum zinc concentration below 60 μg/dL, and zinc status patterns were classified as persistent low zinc, incident zinc deficiency, recovered zinc status, or stable normal zinc status.

Results

Among 267 residents with repeated measurements, persistent zinc deficiency was the predominant pattern and was observed in 184 residents (68.9%). Recovery from zinc deficiency occurred in 14 residents (5.2%), whereas 18 residents (6.7%) developed incident zinc deficiency and 51 residents (19.1%) remained zinc-sufficient throughout follow-up. The prevalence of zinc deficiency changed little between baseline and follow-up, from 198 residents (74.2%) to 202 residents (75.7%), and baseline and follow-up serum zinc concentrations showed a moderate positive correlation.

Conclusions

Low serum zinc concentrations were common and often remained low over time in this cohort of frail long-term care residents. These findings suggest sustained biological or nutritional vulnerability in long-term care settings. However, because serum zinc may be influenced by inflammation, chronic disease, and supplementation, persistent low values should be interpreted cautiously and in conjunction with clinical context and, where possible, additional biomarkers.

## Introduction

Zinc is an essential trace element with fundamental roles in immune regulation, protein synthesis, tissue repair, and cellular metabolism [1-3]. Low zinc status has been associated with impaired host defense, delayed recovery from illness, and increased susceptibility to infection [4-7].

Older adults are especially vulnerable to micronutrient deficiency because inadequate dietary intake, chronic disease, polypharmacy, impaired absorption, and age-related physiological changes may all compromise nutritional status [4,8-10]. Within long-term care facilities, this vulnerability may be amplified by frailty, functional dependence, multimorbidity, and a high burden of malnutrition [9,10].

Several studies have linked low serum zinc concentrations in older adults to adverse clinical outcomes, including pneumonia and impaired immune responsiveness among nursing home residents [5-7]. However, much of the available literature in this population is cross-sectional or interventional, and the natural longitudinal course of zinc deficiency in routine long-term care practice remains poorly characterized [6,11-15].

Accordingly, the aim of this study was to describe the trajectory of serum zinc status among frail residents of a long-term care facility using repeated measurements obtained during routine health examinations over a five-year period.

## Materials and methods

Study design and setting

This longitudinal observational study analyzed routine health examination data from residents of a long-term care facility in Okinawa, Japan, collected between 2021 and 2025.

Data collection

Serum zinc concentrations were measured during annual health examinations at the same health screening center using standardized laboratory methods. The present analysis focused on longitudinal zinc status derived from repeated serum zinc measurements.

Study population

Residents with at least two serum zinc measurements during the study period were eligible for the trajectory analysis.

Definition of zinc deficiency

Zinc deficiency was defined as a serum zinc concentration below 60 μg/dL according to commonly used clinical thresholds [3,12].

Trajectory classification

Serum zinc concentrations were measured annually, with up to five measurements available per resident during the study period. However, for classification purposes, baseline and follow-up values were used to define zinc status patterns consistently across individuals. Residents were classified as having persistent low zinc when both baseline and follow-up concentrations were below 60 μg/dL, incident zinc deficiency when baseline concentrations were 60 μg/dL or greater and follow-up concentrations were below 60 μg/dL, recovered zinc status when baseline concentrations were below 60 μg/dL and follow-up concentrations were 60 μg/dL or greater, and stable normal zinc status when both measurements were 60 μg/dL or greater.

Statistical analysis

Results are summarized descriptively. Zinc status transitions are presented as n (%). The relationship between baseline and follow-up serum zinc concentrations was evaluated using Pearson correlation analysis, and the individual-level distribution was visualized in a scatter plot. The difference in the prevalence of zinc deficiency between baseline and follow-up was evaluated using McNemar’s test for paired proportions. Odds ratios (ORs) with 95% confidence intervals (CIs) were calculated from discordant pairs to estimate the magnitude of transition between zinc deficiency and zinc sufficiency. A two-sided p-value < 0.05 was considered statistically significant.

Sample size considerations

Because this study used routinely collected clinical data from a single long-term care facility, the sample size was determined by the number of residents who had repeated serum zinc measurements during the study period. Therefore, no a priori sample size calculation was performed. However, the analytic cohort included 267 residents with repeated measurements, which was sufficient to describe longitudinal zinc status patterns within this institutional population.

Ethics

This study was approved by the institutional review board of Kubagawa Medical Clinic (Institutional Review Board (IRB) No. KM-2025-07). The requirement for informed consent was waived due to the retrospective nature of the study and the use of anonymized clinical data.

## Results

A total of 267 residents with at least two serum zinc measurements were included in the longitudinal analysis. Low zinc status was common at both observation points, and the transition matrix showed that most residents remained in the same zinc-status category over time (Table 1).

**Table 1:** Baseline-to-follow-up transition in zinc status among residents with repeated measurements (n = 267). Values are presented as number (percentage) of the analytic cohort. Zinc deficiency was defined as a serum zinc concentration <60 μg/dL. Baseline and follow-up categories were determined using the first and last available measurements included in the trajectory analysis. Persistent deficiency indicates zinc <60 μg/dL at both time points; incident deficiency, zinc ≥60 μg/dL at baseline and <60 μg/dL at follow-up; recovery, zinc <60 μg/dL at baseline and ≥60 μg/dL at follow-up; and stable normal status, zinc ≥60 μg/dL at both time points.

Baseline zinc status	Follow-up zinc <60 μg/dL, n (%)	Follow-up zinc ≥60 μg/dL, n (%)	Total, n (%)
Baseline zinc <60 μg/dL	184 (68.9%)	14 (5.2%)	198 (74.2%)
Baseline zinc ≥60 μg/dL	18 (6.7%)	51 (19.1%)	69 (25.8%)
Total	202 (75.7%)	65 (24.3%)	267 (100.0%)

At baseline, 198 residents (74.2%) met the definition of zinc deficiency. Of these, 184 of 198 residents (92.9%) remained deficient at follow-up, whereas only 14 of 198 residents (7.1%) crossed above the deficiency threshold. Among 69 residents (25.8%) with baseline zinc concentrations of 60 μg/dL or greater, 51 of 69 residents (73.9%) remained zinc-sufficient, and 18 of 69 residents (26.1%) developed incident deficiency. Accordingly, the cohort prevalence of zinc deficiency changed little between baseline and follow-up, increasing slightly from 198 residents (74.2%) to 202 residents (75.7%). The difference in zinc deficiency prevalence between baseline and follow-up was not statistically significant (McNemar’s test, p = 0.48). The OR for worsening zinc status compared with recovery was 1.29 (95% CI 0.64-2.58).

**Figure 1:**
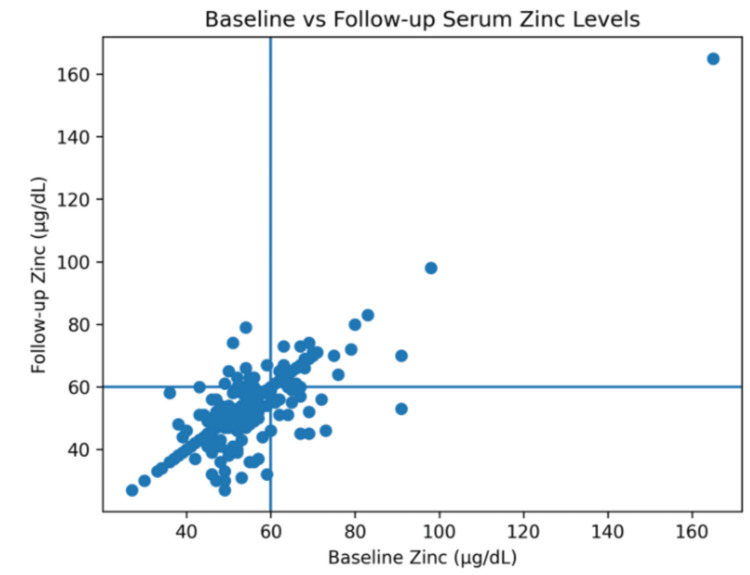
Relationship between baseline and follow-up serum zinc concentrations among long-term care facility residents

The individual-level scatter plot further demonstrated the relationship between baseline and follow-up serum zinc concentrations (Figure 1). Most observations clustered near the deficiency threshold, with a dense concentration of points in the lower-left quadrant, indicating that residents who started with low zinc concentrations generally remained below 60 μg/dL at follow-up. Only a small number of residents showed clearly higher zinc concentrations at either time point. Baseline and follow-up serum zinc concentrations showed a moderate positive correlation (Pearson correlation coefficient r = 0.51, p = 0.0004). A fitted linear regression line further illustrated the positive association between the two measurements.

Scatter plot illustrating the relationship between baseline and follow-up serum zinc levels among residents with repeated measurements. Each point represents an individual resident. The solid line represents the fitted linear regression line. Baseline and follow-up zinc concentrations were moderately correlated (Pearson correlation coefficient r = 0.51, p = 0.0004).

## Discussion

This five-year longitudinal analysis showed that low serum zinc concentrations were common among residents of this long-term care facility and frequently persisted from baseline to follow-up. Continued low serum zinc was the most common classification pattern, whereas apparent recovery was relatively uncommon, and a smaller subgroup developed incident low zinc during follow-up. Taken together, the transition matrix and scatter plot suggest that repeated low serum zinc values in this setting are often sustained rather than isolated findings.

These observations are clinically relevant because zinc has important roles in immune regulation, protein synthesis, tissue repair, and broader metabolic homeostasis, and low zinc status in older adults has been associated with impaired host defense and increased susceptibility to infection [1-8]. At the same time, serum zinc concentration alone does not always confirm true nutritional zinc deficiency, particularly in older adults with chronic disease. Inflammation, multimorbidity, altered protein status, infection, and other physiological changes common in frail long-term care populations may influence circulating zinc levels [4,8-10]. Accordingly, persistent low serum zinc in this cohort should not be interpreted as definitive evidence of nutritional deficiency in isolation, but rather as a clinically meaningful finding that should be considered together with the overall clinical context and, where available, additional nutritional or inflammatory markers.

The present findings also extend previous literature in older adults, much of which has been cross-sectional or focused on supplementation, by suggesting that low serum zinc may remain stable over time under routine long-term care conditions [5-7,11]. From a practical standpoint, serial zinc monitoring may help identify residents with sustained biological or nutritional vulnerability who warrant closer clinical evaluation. In facilities caring for highly frail populations, repeated low serum zinc values may therefore serve as a pragmatic screening signal, even if they do not by themselves establish the etiology of the abnormality.

Another important consideration is that a small number of residents received zinc supplementation during the study period. Because detailed longitudinal data regarding the timing, dose, duration, and adherence of supplementation were not systematically available, the present analysis cannot clearly distinguish the natural course of low serum zinc from treatment-related change. Supplementation may have contributed to apparent recovery in some residents and may have attenuated the persistence of low serum zinc observed over time. This should be taken into account when interpreting the reported patterns.

Limitations

This study has several limitations. First, it was conducted in a single long-term care facility in Okinawa, which may limit generalizability to other institutional settings. Second, although serum zinc was measured annually and up to five measurements were available per resident during the study period, the present classification used baseline and follow-up values to define zinc status patterns consistently across individuals. The reported categories, therefore, represent a simplified two-point classification rather than a full longitudinal trajectory incorporating all intermediate measurements. Third, detailed data on dietary zinc intake, inflammatory markers, medication exposure, and zinc supplementation were not systematically available, which limited evaluation of the determinants of persistent low serum zinc and prevented a clear distinction between true nutritional deficiency and inflammation-related or treatment-related changes. Finally, the observational design does not permit causal inference.

Future studies should combine serial zinc assessment with inflammatory markers, nutritional measures, and systematic documentation of supplementation exposure to better define the causes and clinical significance of persistent low serum zinc in long-term care residents.

## Conclusions

In this cohort of frail long-term care residents, low serum zinc concentrations were common at baseline and frequently remained low at follow-up. These findings suggest that repeated low serum zinc values may reflect sustained biological or nutritional vulnerability in long-term care settings. However, because serum zinc can be influenced by inflammation, chronic disease, and supplementation, persistent low values should be interpreted cautiously and not attributed to nutritional deficiency on the basis of serum zinc alone. Longitudinal monitoring may still be useful as part of a broader clinical assessment, especially when combined with additional nutritional and inflammatory indicators.
